# Depressive Tendency Biases Ensemble Perception of Emotional Faces

**DOI:** 10.1155/da/9996485

**Published:** 2025-11-17

**Authors:** Zhen Lin, Mingliang Gong, Yufei Chen, Han Sheng

**Affiliations:** ^1^School of Psychology, Jiangxi Normal University, Nanchang, China; ^2^School of Public Administration and Emergency Management, Jinan University, Guangzhou, China

**Keywords:** depressive tendencies, emotion, ensemble perception, exposure time, negative bias

## Abstract

Previous studies have shown that individuals with depression have an impaired ability in encoding single facial expressions. However, little is known about how depressive tendencies—subclinical emotional distress that may progress to clinical depression—affect the perception of the average emotion of multiple faces. To address this question, the current study investigated whether depressive tendencies affect explicit or implicit ensemble perception of emotion. In Study 1, participants viewed sets of four emotionally varying faces (ranging from angry to happy) for 2000 or 50 ms, then judged if a subsequent test face was angrier than the average emotion of the preceding set. Results showed that the high depressive symptom (HDS) group had a point of subjective equality (PSE) more biased toward anger compared to the low depressive symptom (LDS) group when exposure time was 2000 ms. However, this difference disappeared when the time was shortened to 50 ms. In Study 2, we assessed the automatic perception of ensemble emotion by requiring participants to judge whether a probe face was a member of the preceding set, a task that does not explicitly demand averaging. Results indicated that the HDS and LDS groups had a similar likelihood of misidentifying the set mean as a member under both 2000 and 50 ms conditions, indicating a comparable automatic coding of ensemble emotion. Together, the current research demonstrates that depressive tendencies can bias ensemble coding for emotional faces at explicit level but not at implicit level.

## 1. Introduction

The ability to perceive emotional facial expressions is critical for understanding a person's intentions, thoughts, and emotions (e.g., [1]). In complex social settings, such as giving a public speech, however, people commonly interact with multiple individuals at the same time [2]. Notably, people can spontaneously extract a summary statistical representation from a set of faces, known as ensemble perception or ensemble coding. The human visual system has a tendency to represent a set of similar objects as summary statistics, rather than encoding each item individually [3–7].

Ensemble perception has been reported across various types of stimuli, ranging from low-level visual features to mid- and high-level visual information [7]. This includes orientation [8, 6], speed [9], average size in a set of circles [4, 10], and average gender or facial identity of a crowd [11, 12], et cetera. An increasing number of studies in recent years have keenly focused on the ensemble perception of high-level objects, such as emotions [13–15], biological motion [16], animacy of objects [17], trustworthiness [18], and semantic meaning of numerical value [19]. Haberman and Whitney [5, 20], for example, asked participants to assess the mean facial expression from a set of faces varying in emotionality (e.g., happy–sad) and found that observers could rapidly and accurately evaluate the mean emotional expression of the face set. These findings demonstrate that humans are sensitive to the summary statistical information in a crowd of faces [17, 20–22].

While prior research has mainly focused on the influence of emotional states on perception of emotions expressed by individual faces [23, 24], the perception of face ensemble can also be influenced by the emotional states of observers. Peng et al. [25] assessed visual averaging performance using a membership identification task in which a set of four face identities was shown briefly, followed by a probe face (including the morphed average of the preceding set). Participants judged whether the probe face was a member of the preceding set, with their visual averaging performance indexed by the tendency to treat the set's average face as a member. They found that participants tended to choose the average of face identity increased when positive emotions were induced via watching movie clips, whereas their performance decreased after negative emotions were induced. This finding has also been demonstrated in people with emotional disorders, including those with autism [26, 27] and social anxiety [2, 28, 29]. For example, compared to people with lower levels of social anxiety, those with high levels of social anxiety perceive the overall facial emotion of crowds more negatively (e.g., [28]).

To our knowledge, however, no research has yet investigated how depressive tendencies—a prevalent subclinical state characterized by significant emotional distress and functional impairment—affect the perception of ensemble emotion. Individuals with depressive symptoms include those clinically diagnosed with depression and those with depressive tendencies. While not as severe as depression, individuals with depressive tendencies also experience significant emotional distress and functional impairment, such as persistent sadness, feelings of loss, and self-denial. If these symptoms persist and have a significant impact on daily functioning and personal life, then this condition may further develop into depression [30]. Research suggests that both individuals with clinical depression [23, 24, 31] and individuals with depressive tendencies [32] exhibit an attentional bias toward negative or threat-related emotional cues while having a reduced attention to positive faces. Critically, these emotion perception deficits constitute core manifestations of depression's negative cognitive bias [33]. Investigating such perceptual biases in individuals with depressive tendencies may contribute to the refinement of cognitive models of depression. Furthermore, as depressive tendencies represent the prodromal stage of clinical depression [30], the findings of the present study may provide objective indicators for early screening of depression. Additionally, previous studies have predominantly focused on the processing of individual faces (e.g., [23, 24, 32]), while relatively little is known about how depressive tendencies affect the processing of multiple emotional faces. However, in real-world social interactions, individuals often need to rapidly integrate emotional cues from multiple faces simultaneously [20]. Studies examining single-face emotion perception cannot adequately capture this complexity.

The purpose of this study was to explore the impact of depressive tendencies on the ensemble perception of multiple faces. To this end, our two studies employed two paradigms commonly used in the study of ensemble perception. In Study 1, participants were first presented with a set of emotional faces, and they were explicitly instructed to evaluate whether a subsequently presented probe face was angrier than the average emotion of the face set. We focused on participants' point of subjective equality (PSE) and response accuracy. The PSE is a classic psychophysical index widely used in ensemble perception studies of spatial size [34], numerical value [19], and temporal duration [34, 35]. It refers to the value at which an individual perceives a test stimulus as being identical to a standard stimulus, even if their physical properties differ. The PSE helps researchers understand how stimuli are perceived, detect perceptual biases, and establish sensory thresholds across visual, auditory, and other sensory modalities. In Study 2, a different paradigm called membership identification was employed. In the paradigm, a set of emotional faces was firstly presented to participants. Following this, they were instructed to judge whether a subsequently presented probe face was a member of the previous face set—with no instruction to estimate the average emotion of the set. A consistent finding across prior studies is that participants often misidentify the “mean face” (which was not a real member) as a member of the set [20, 22, 36, 37]. This phenomenon indicates the spontaneous formation of an ensemble representation, reflecting implicit and automatic ensemble perception. In both studies, the exposure time of the face sets was also manipulated. Research has shown that negative bias is evident in depressed patients when stimuli are presented for longer durations (e.g., 1000 ms) [38–40], but not for shorter durations (e.g., 14 ms) [41]. Findings also suggest that the length of exposure time affects the perception of facial expression ensembles (e.g., [22]). We hypothesized that relative to the low depressive symptom (LDS) group, the high depressive symptom (HDS) group would exhibit a deficit in ensemble perception. Specifically, in Study 1, the PSE of the HDS group would be more biased towards negative emotions and demonstrate lower accuracy in explicit ensemble perception than the LDS group. In Study 2, the HDS group would be less likely to automatically engage in ensemble perception of emotions than the LDS group. We also expected that these results would only occur when the faces were presented for the longer duration.

## 2. Study 1A

This study was exploratory. We first explored the performance in explicit ensemble perception of emotion between the HDS group and LDS group when exposure time was 2000 ms.

### 2.1. Method

#### 2.1.1. Participants

Following the procedure of Li et al. [36], we utilized G*⁣*^*∗*^Power 3.1 software [42] to determine the minimum sample size required for two two-factor mixed designs. While the present study included three independent variables, the design varied according to dependent variables. For percentage of “angrier” responses, it was a 7 (distance from the mean: −12 vs. −8 vs. −4 vs. 0 vs. 4 vs. 8 vs. 12) × 2 (group: HDS vs. LDS) mixed design, while for accuracy rate, it was a 2 (test face: anger vs. happiness) × 2 (group: HDS vs. LDS) mixed design. The analysis revealed that a minimum of 22 and 46 participants were needed, respectively, assuming a priori medium effect size (*f*) of 0.25, a statistical test power (1 – *β*) of 0.9, and a significance level (*α*) of 0.05. Since participants were divided into two groups according to depression scale scores, at least 23 participants were required for each group. Sixty-three undergraduate students from Jiangxi Normal University (26 men, 37 women; mean age = 20.1 years old) participated in exchange for money or course credits. They consisted of the HDS group (11 men, 19 women, mean age = 19.9 years old) and LDS group (15 men, 18 women, mean age = 20.3 years old). Participants were naive to the purpose of the experiment and had normal or corrected-to-normal vision. Informed consent was obtained from all participants before running the study. The study was approved by the Review Board of School of Psychology, Jiangxi Normal University.

The procedure for screening subjects for depressive tendencies was as follows. First, the Chinese version of the Center for Epidemiologic Studies Depression Scale (CES-D; [43]) was used to assess participants' levels of depression in the general population. The CES-D was originally developed by Radloff [44] and has demonstrated good reliability and validity when applied to Chinese students [45, 46]. It consists of 20 items (e.g, “I was bothered by things that usually don't bother me”), with a score ranging from 0 (occasional or none) to 3 (most of the time or continuous) on a Likert-type scale that asks for frequency of symptoms associated with depression in the last week. A total score of 16 points or more is regarded to have a high depressive tendency. The Cronbach's *α* of CES-D was 0.90 in the present study. Then, on the day of experiment, participants' levels of depression were reassessed using the Chinese version of the Self-rating Depression Scale (SDS; [47]). The scale consists of 20 items (e.g, “I feel down-hearted and blue”), with a score ranging from 1 (rarely) to 4 (most of the time). According to the Chinese norm, the total score of 42 points or more is regarded to have a high depressive tendency. The Cronbach's *α* of SDS was 0.88 in the present study.

The correlation coefficient between the two scales is 0.90. Only participants who met the depression criteria of both scales simultaneously were selected as the HDS group, while the others were in LDS group. In the current study, the mean CES-D score (27.00, SD = 5.71) and the mean SDS score (48.03, SD = 3.54) in HDS group were significantly higher than those (*M* = 10.94, SD = 5.56; *M* = 33.88, SD = 5.57, respectively) in LDS group, *t*(1,61) = 11.31, *p* < 0.001, *d* = 2.85; *t* (1,61) = 11.91, *p* < 0.001, *d* = 3.03. There were no significant differences between the two groups in age and gender (*ps* > 0.05).

#### 2.1.2. Stimuli and Apparatus

From the Chinese Facial Affective Picture System (CFAPS; [48]), two faces of the same model displaying two emotional expressions (i.e., happiness and anger) were chosen. We chose angry faces as the representative of negative expressions because the biased ensemble perception of angry expressions has been associated with heightened depression levels [49]. Each cropped face image was standardized in size (2.8° in width and 3.2° in height). The intensity of these two emotions was adjusted using FaceFilter Studio 2. Then 21 college students (11 men and 10 women) who did not participate in the formal experiment were additionally recruited to rate the emotional valence and arousal of the two faces. Arousal referred to how angry or happy faces made one feel at the physiological level. They first determined whether the emotion of the face was angry or happy. Then they rated the emotional arousal of the faces on a 7-point Likert scale ranging from 0 (extremely calm) to 7 (extremely excited). Results showed that the mean arousal scores were 5.38 (SD = 1.20) for the angry expression, 5.19 (SD = 0.51) for the happy expression, respectively. Using paired-samples *t*-tests, no significant differences were found between the two expressions in arousal, *t*(1,20) = 0.81, *p* = 0.428.

**Figure 1:**
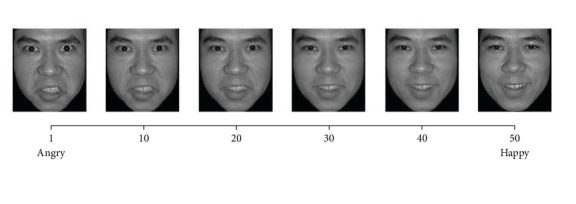
Schematic illustration of 50 morphed faces with varying emotional intensity ranged from anger to happiness.

Following Haberman and Whitney [20], we created 50 morphed faces with varying emotional intensity ranging from anger to happiness using Abrosoft FantaMorph 5 (Figure 1). Each morphed face corresponded to one of 50 emotional units, with Face 1 being the angriest and Face 50 being the happiest. Face N (1 ≤ *N* < 50) was always one emotional unit angrier than faces *N* + 1. The larger the emotional units between two faces, the easier it is to distinguish between them.

During the experiment conducted using E-prime 2.0, the face stimuli were presented on a 19-inch computer screen with a black background. The screen had a resolution of 1920 × 1200 pixels and a refresh rate of 60 Hz. Participants were seated 60 cm in front of the screen.

#### 2.1.3. Design and Procedure

The experiment used a 2 (test face: anger vs. happiness) × 7 (distance from the mean: −12 vs. −8 vs. −4 vs. 0 vs. 4 vs. 8 vs. 12) × 2 (group: HDS vs. LDS) mixed design. The variables of test face and distance from the mean were within-subject factors, and seven levels meant that the emotion of the test face could be 0, ± 4, ± 8, or ± 12 emotional units away from the mean emotion of the set of four faces. The variable of group was a between-subject factor. The PSE and the accuracy of the ensemble perception of emotional intensity served as the dependent variables.

**Figure 2:**
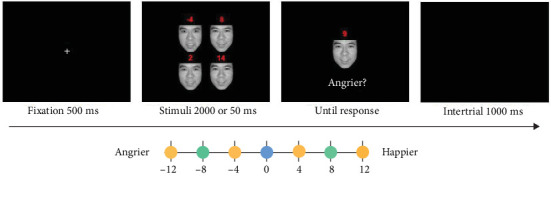
Schematic illustration of a typical trial in Study 1. Stimuli were presented for a duration of 2000 ms in Study 1A and 50 ms in Study 1B. The numbers positioned above the faces signify emotional intensity of each face relative to the mean face, and they were not seen by participants. The test face can be any distance from the mean emotion of the face set. The number “0” indicates the mean test face of the face set. The positive numbers indicate the happier test faces relative to the face set. The negative numbers indicate the angrier test faces relative to the face set.

As shown in Figure 2, each trial started with a fixation cross that appeared in the center of the screen for 500 ms. Next, a set of four faces, each with a different level of emotion, was displayed in the center, forming a 2 × 2 matrix (8.2° in width and 8.6° in height). Before each trial, the mean emotional intensity of the set of faces was randomly selected from 14 to 37. Once the mean emotion was determined, four corresponding emotional faces were generated, with two of them being angrier than the mean emotional intensity (mean − 3 and mean − 9) and the other two being happier (mean + 3 and mean + 9). This resulted in a minimum distance of 6 emotion units between any two of the four faces—a value that is typically well above participants' perceptual discrimination thresholds [20] and has been widely adopted in prior ensemble perception studies [5, 14, 20, 22]. The four faces were presented for 2000 ms, followed by a test face that appeared in the center of the screen until a response was made. Participants were instructed to determine whether the test face appeared angrier than the mean emotion of the preceding set of faces, and indicate their response by pressing either the “S” or “K” button on the keyboard. The response keys were counterbalanced across participants. While the response speed was not required, participants were asked to respond as accurately as possible. A blank screen was displayed for 1000 ms after participant's response, and then the next trial began.

Each participant completed 20 practice trials and 560 experimental trials in total. To lessen the impact of fatigue, participants were required to take a break after every 180 trials.

### 2.2. Results and Discussion

**Figure 3:**
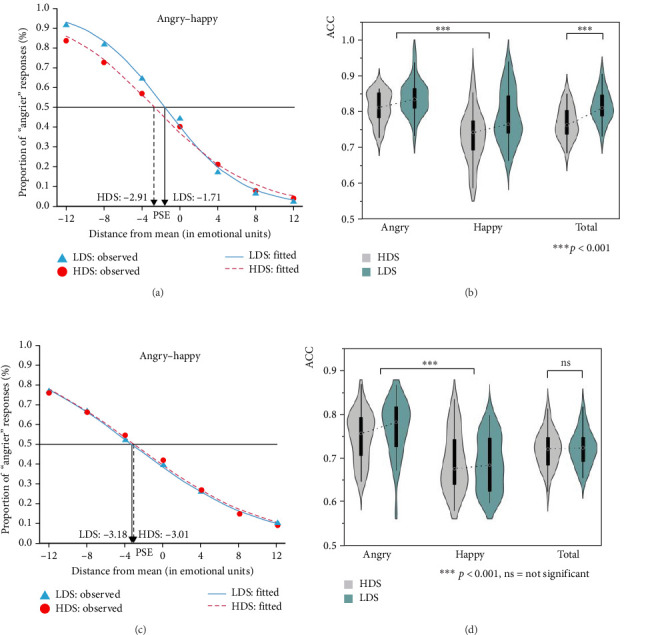
Results of Studys 1A (a and b) and 1B (c and d). The top panels show the point of subjective equality (PSE; a) and the ensemble perceptual accuracy of recognizing facial expressions (b) for groups of HDS and LDS when exposure time was 2000 ms in Study 1A. The bottom panels represent the PSE (c) and the ensemble perceptual accuracy (d) for the two groups when exposure time was 50 ms in Study 1B.

One participant of the HDS group was excluded from the data set due to low accuracy (<0.500). The overall mean reaction time for the remaining participants was 1508.79 ms, with a standard deviation of 730.48 ms. Trials with response times exceeding two standard deviations from the mean were excluded from further analysis, which accounted for 2.38% of the total trials. Results are plotted in Figure 3a,b.

Following Wang and Jiang [50], the data for each participant were fitted with the Boltzmann sigmoid function Equation (1) (*R*^2^ was 0.888–0.998 and 0.872–0.997 for the HDS group and LDS group, respectively), using the nonlinear fitting tool in OriginPro 2021 software. Based on this function, we calculated the PSE for HDS group and LDS group—the point on the 50% threshold where the emotionality of the test face had a 50% chance of being perceived as angrier than the average emotion of the previous set of faces, and a 50% chance of being perceived as less angry. As the curve showed in Figure 3a, when the proportion of “angrier” responses is equal to 0.5 (i.e., when 50% of the subjects' responses are “negative”), the point corresponding to this value on the abscissa is the PSE. A PSE value lower than 0.5 means that the participants perceive the mean emotion of face crowds as more negative.

(1) $$\begin{array}{c} f\left(x\right)=1/\left(1+\;\exp\left[\left(x-x0\right)/\omega \right]\right)\;. \end{array}$$

First, a one-sample *t*-test was used to examine whether there was a significant difference between the two groups' PSE and 0 (no emotional bias). The results indicated that the PSE of both HDS and LDS groups was significantly smaller than 0, *t*(1, 29) = −5.93, *p* < 0.001, *d* = 1.08; *t*(1, 32) = −5.57, *p* < 0.001, *d* = 0.97. Next, an independent samples *t*-test was conducted to analyze the group differences in the PSE. The results showed that HDS group had a significantly lower PSE (*M* = −2.91, SD = 2.69) than the LDS (*M* = −1.71, SD = 1.76), *t* (1,61) = −2.11, *p* = 0.04, *d* = 0.53. This finding suggests that compared to the LDS group, the HDS group tends to perceive ambiguous facial expressions as angrier, indicating a more pronounced negative emotional bias in their ensemble perception. Finally, a 7 (distance from the mean: −12 vs. −8 vs. −4 vs. 0 vs. 4 vs. 8 vs. 12) × 2 (group: HDS vs. LDS) conditions mixed repeated-measures analysis of variance (ANOVA) was conducted on the proportion of “angrier” responses. Since the assumption of sphericity was violated in Mauchly's Test, *χ*^2^ (20) = 223.67, *p* < 0.001, Greenhouse–Geisser corrections were applied. Results showed a significant two-way interaction, *F*(2.54, 154.72) = 9.14, *p* < 0.001, *η*_p_^2^ = 0.13. The simple effect analysis revealed significant group differences at the distance from the mean of −12 (HDS: *M* = 0.836 vs. LDS: *M* = 0.916, *p* = 0.001), −8 (HDS: *M* = 0.727 vs. LDS: *M* = 0.817, *p* = 0.003), and −4 (HDS: *M* = 0.569 vs. LDS: *M* = 0.643, *p* = 0.028), which were consistent with the finding that the HDS group was more likely to perceive the average facial emotion as angrier. The main effect of distance from the mean was significant, *F*(2.54, 154.72) = 1469.76, *p* < 0.001, *η*_p_^2^ = 0.96. The main effect of group was not significant, *F*(1, 61) = 3.18, *p* = 0.080, *η*_p_^2^ = 0.05.

Furthermore, a mixed repeated-measures ANOVA was conducted on the accuracy of recognizing facial expressions (anger vs. happiness) for the two groups (Figure 3b). The results showed no significant interaction effect between test face type and group, *F*(1, 61) = 1.51, *p* = 0.223, *η*_p_^2^ = 0.02. The main effect of test face type was significant, *F*(1, 61) = 35.31, *p* < 0.001, *η*_p_^2^ = 0.37. Specifically, the accuracy of recognizing angry facial expressions (*M* = 0.824, SD = 0.006) was significantly higher than that of recognizing happy facial expressions (*M* = 0.760, SD = 0.009), indicating an anger processing advantage when judging average emotional expressions. The main effect of group was also significant, *F*(1, 61) = 14.81, *p* < 0.001, *η*_p_^2^ = 0.20. Specifically, the ensemble perceptual accuracy in HDS group (*M* = 0.771, SD = 0.008) was significantly lower than that in LDS group (*M* = 0.813, SD = 0.007), which revealed that the perceptual ability of ensemble emotion was impaired for people with depressive tendency under the condition of 2000 ms exposure time.

**Figure 4:**
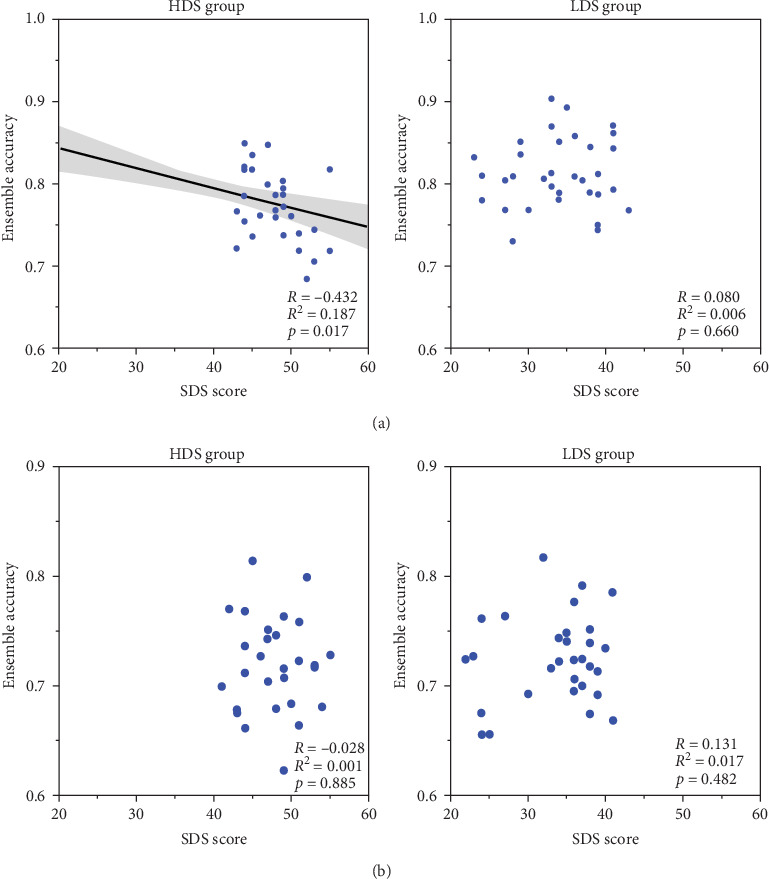
The correlation between depressive tendency and ensemble perception accuracy within each group separately when exposure time was 2000 ms in Study 1A (a) and when exposure time was 50 ms in Study 1B (b).

To further explore the relationship between high depressive tendency and the accuracy on ensemble perception under the condition of 2000 ms exposure time, the correlation analyses were conducted within each group separately (Figure 4a). For HDS group, the result showed a significant negative correlation between them, *r* (30) = −0.432, *p* = 0.017, whereas for LDS group, no significant correlation was found, *r* (33) = 0.080, *p* = 0.660.

In summary, Study 1A showed a significant difference between HDS and LDS group in explicit ensemble perceptual judgment of emotional faces when exposure time was 2000 ms, with HDS group having a more negative bias. Mogg et al. [41] showed that negative attentional bias was not present in individuals with depression when the stimulus presentation time was short. We postulate that the exposure time for facial stimuli might serve as a boundary condition for this underlying mechanism. Next, in Study 1B, we shortened the stimulus presentation time to observe whether there was a significant difference between HDS and LDS group in the ensemble perceptual performance. Previous research has established that robust ensemble representations can be formed from stimulus sets presented for as little as 50 ms [20, 22, 51], a duration that precludes detailed processing of individual items [22]. Following these established protocols, we employed a 50 ms exposure time in Study 1B.

## 3. Study 1B

### 3.1. Method

#### 3.1.1. Participants

Sixty-one undergraduate students from Jiangxi Normal University (23 men, 38 women; mean age = 20.52 years old) participated in exchange for money or course credits. They consisted of the HDS group (11 men, 19 women, mean age = 20.03 years old) and LDS group (13 men, 18 women, mean age = 21.00 years old). Participants were naive to the purpose of the experiment and had normal or corrected-to-normal vision. Informed consent was obtained from all participants before running the study. The study was approved by the Review Board of School of Psychology, Jiangxi Normal University.

In this experiment, the mean CES-D score (28.13, SD = 5.48) and the mean SDS score (47.77, SD = 3.73) in HDS group were significantly higher than those (*M* = 10.39, SD = 5.33; *M* = 33.81, *SD* = 5.87, respectively) in LDS group, *t*(1,59) = 12.82, *p* < 0.001, *d* = 3.28 and *t*(1,59) = 11.05, *p* < 0.001, *d* = 2.84, respectively. There were no significant differences between the two groups in age or gender (*ps*  > 0.05).

#### 3.1.2. Stimuli and Apparatus

The stimuli and apparatus were the same as those used in Study 1A.

#### 3.1.3. Design and Procedure

The design and procedure were identical to those of Study 1A except that the exposure time for the face stimuli in this experiment was decreased to 50 ms.

### 3.2. Results and Discussion

Similar analyses as Study 1A were conducted for Study 1B, and results are plotted in Figure 3c,d. We plotted the scale of the “angrier” response and fitted the data for each participant in two groups with the Boltzmann sigmoid function.

First, two separate one-sample *t*-tests were used to examine whether there were significant differences between the two groups' PSEs and 0 (no emotional bias). The results indicated that the PSE of both HDS and LDS groups was significantly smaller than 0, *t*(1, 29) = −4.58, *p* < 0.001, *d* = 0.84, and *t*(1, 30) = −5.28, *p* < 0.001, *d* = 0.95, respectively. Next, an independent samples *t*-test was conducted to analyze the group differences in the PSE. No significant differences were found between HDS group (*M* = −3.01, SD = 3.60) and LDS group (*M* = −3.18, SD = 3.36), *t*(1, 59) = 0.20, *p* = 0.84, which suggested that compared with LDS group, HDS group did not show a significant negative emotional bias when perceiving the mean emotion of the set of faces. Finally, a mixed repeated-measures ANOVA was conducted on the proportion of “angrier” responses. Since it violated the prerequisite of Mauchly's Test of Sphericity, *χ*^2^ (20) = 148.21, *p* < 0.001, Greenhouse–Geisser corrections were applied. There was no significant interaction between distance from the mean and group, *F*(2.60, 153.50) = 0.76, *p* = 0.499, *η*_p_^2^ = 0.01. The main effect of distance from the mean was significant, *F*(2.60, 153.50) = 872.91, *p* < 0.001, *η*_p_^2^ = 0.94. The main effect of group was not significant, *F* (1,59) = 0.04, *p* = 0.849, *η*_p_^2^ < 0.001.

Furthermore, a mixed repeated-measures ANOVA was conducted on the accuracy of recognizing facial expressions (anger vs. happiness) in the two groups. The results showed no significant interaction effect between test face type and group, *F* (1, 59) = 0.31, *p* = 0.581, *η*_p_^2^ = 0.01. The main effect of test face type was significant, *F*(1, 59) = 26.89, *p* < 0.001, *η*_p_^2^ = 0.31. Specifically, the accuracy of recognizing angry facial expressions (*M* = 0.755, SD = 0.008) was significantly higher than that of recognizing happy facial expressions (*M* = 0.689, SD = 0.008). The main effect of group was not significant, *F*(1,59) = 0.05, *p* = 0.817, *η*_p_^2^ < 0.01.

Likewise, we conducted a correlation analysis between the scores of depression and the accuracies of ensemble perception within each group separately (Figure 4b). The results showed no significant correlation, for HDS group, *r* (30) = −0.028, *p* = 0.885, and for LDS group, *r* (31) = 0.131, *p* = 0.482.

In summary, Study 1B showed that both HDS and LDS groups showed better recognition performance for the mean emotion of anger, even when presented with stimuli for an extremely short duration of just 50 ms, which reflected the anger superiority effect. Moreover, when exposure time of the set faces was 50 ms, there was no significant difference between HDS group and LDS group in their performance of ensemble perception. This finding suggests that under the condition of 50 ms exposure time, HDS group's ability to represent ensemble emotion is not impaired.

## 4. Study 2

In Study 1, we explored the effect of depressive tendency on ensemble perception when participants were explicitly asked to judge the ensemble emotion of the set faces. Numerous studies have demonstrated that ensemble perception can be formed implicitly and automatically [20, 22, 36, 37]. These studies employed a membership identification task in which participants were only instructed to judge whether the test face was a member of the preceding face set, without any instruction to estimate the average emotion. The consistent finding is that participants often misidentify the “mean face” (which was not an actual member) as a member of the set. This phenomenon indicates the spontaneous formation of an ensemble representation, reflecting implicit and automatic ensemble perception. In Study 2, we used this membership identification task to investigate the potential implicit effects of depressive tendencies on ensemble perception in the absence of explicit judgment demands.

### 4.1. Method

#### 4.1.1. Participants

The design of Study 2 differed from Study 1, and it was a 3 (test face type: mean face vs. member face vs. neither face) × 2 (exposure time: 50 ms vs. 2000 ms) × 2 (group: HDS vs., LDS) mixed design, with test face type and exposure time as within-subject factors. Since G*⁣*^*∗*^Power is not applicable for calculating sample sizes involving two or more within-subject independent variables, this experiment did not use G*⁣*^*∗*^Power to estimate required sample size. However, the sample size was kept consistent with that of Study 1A and 1B. Fifty-eight undergraduate students from Jiangxi Normal University (22 men, 36 women; mean age = 20.29 years old) participated in the experiment for an exchange of money or course credits. They consisted of the HDS group (11 men, 16 women, mean age = 19.81 years old) and LDS group (11 men, 20 women, mean age = 20.71 years old). Participants were naive to the purpose of the experiment and had normal or corrected-to-normal vision. Informed consent was obtained from all participants before running the study. The study was approved by the Review Board of School of Psychology, Jiangxi Normal University.

In this experiment, the mean CES-D score (26.11, SD = 5.16) and the mean SDS score (46.89, SD = 3.29) in HDS group were significantly higher than those (*M* = 10.42, SD = 5.66; *M* = 33.90, SD = 5.75, respectively) in LDS group, *t*(1,56) = 10.97, *p* < 0.001, *d* = 2.90 and *t*(1,56) = 10.35, *p* < 0.001, *d* = 2.77, respectively. There were no significant differences between the two groups in age or gender (*ps*  > 0.05).

#### 4.1.2. Stimuli and Apparatus

The stimuli and apparatus were the same as those used in Study 1, comprising morphed faces from the same model with varying emotional intensities.

#### 4.1.3. Design and Procedure

**Figure 5:**
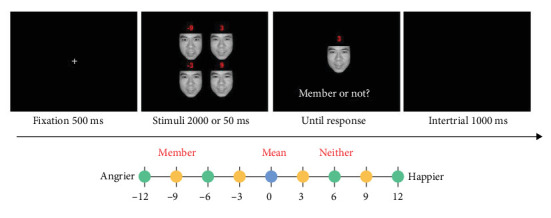
Schematic illustration of a trial in Study 2. The test face can be represented by the circles at any given distance. The blue circle indicates the mean test face of the face crowd. The orange circles indicate the member test faces of the face crowd. The green circles indicate the neither test faces of the face crowd.

As illustrated in Figure 5, each trial began with a fixation cross presented in the center of the screen for 500 ms. Then a set of four faces with varying emotional units (the same as in Study 1) for 50 or 2000 ms, followed by a test face that appeared in the center of the screen. The test face could be: (i) the mean face (the face that had the mean emotional intensity of the preceding set); (ii) a member face (a member of the preceding set); (iii) a “neither” face (a face that is neither the mean face nor the member face). Participants were instructed to determine whether the test face was a member of the preceding face set by pressing the button of “S” and the button of “K” on the keyboard, respectively. Other procedures were consistent with Study 1A.

Exposure time (i.e., 50 and 2000 ms) was manipulated in blocks throughout the entire experiment. Each block consisted of 20 practice trials and 216 experimental trials, and trials in each block were presented randomly. To lessen the impact of fatigue, participants were required to take a break after every 108 trials.

### 4.2. Results and Discussion

**Figure 6:**
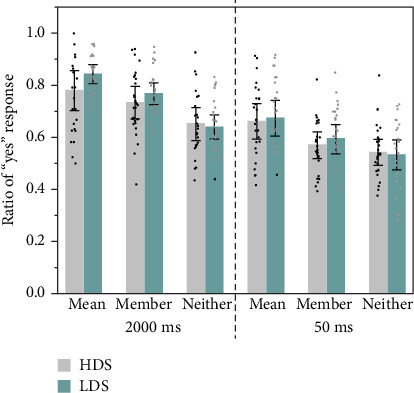
The ratio of “yes” responses for three types of faces in groups of HDS and LDS at a stimulus presentation of 2000 and 50 ms. Error bars correspond to one standard error.

Trials with response times beyond two standard deviations of the overall mean were excluded from further analyses, accounting for 2% of all trials. Following the analytic procedure of Haberman and Whitney [20], we analyzed the proportion of “yes” responses. Results are plotted in Figure 6. A “yes” response indicated that the observers thought that the test face was a member face.

A 3 (test face type: mean face vs. member face vs. neither face) × 2 (exposure time: 50 ms vs. 2000 ms) × 2 (group: HDS vs. LDS) repeated-measures ANOVA revealed that there was no significant three–way interaction, *F*(2, 55) = 1.09, *p* = 0.340, *η*_p_^2^ = 0.02. The interaction between the test face type and exposure time was significant, *F*(2, 56) = 8.00, *p* = 0.001, *η*_p_^2^ = 0.13. Simple effect analysis showed that regardless of stimulus presentation time, the proportion of “yes” judgment for the mean face was significantly higher than that for the member face and neither face (*ps* < 0.001), and the proportion of member face was significantly higher than that of neither face (*p* < 0.001). For the mean face, the proportion of “yes” judgment at the presentation of 2000 ms was significantly higher than that at the presentation of 50 ms (*p* < 0.001). This result indicated that exposure time had a significant impact on ensemble perceptual performance. The interaction effect between the type of face stimuli and the group was not significant, *F*(2, 56) = 2.01, *p* = 0.139, *η*_p_^2^ = 0.04, either was the interaction effect between the stimulus presentation time and the group significant, *F*(2, 56) = 0.54, *p* = 0.464, *η*_p_^2^ = 0.01.

The main effect of the type of test face was significant, *F*(2, 56) = 143.74, *p* < 0.001, *η*_p_^2^ = 0.72. Multiple comparisons with Bonferroni correction showed that the proportion of “yes” judgments for the mean face (*M* = 0.781, SD = 0.013) was significantly higher compared to the member face (*M* = 0.656, SD = 0.013) (*p* < 0.001), and both the mean and member faces were significantly higher than the neither face (*M* = 0.560, SD = 0.014) (*ps* < 0.001). These results indicated that although being instructed to focus on the emotions of individual members in each set of faces, both the HDS and LDS groups implicitly coded the ensemble and tended to misidentify the mean face as a member of the face set. The main effect of stimulus presentation time was significant, *F*(1, 56) = 11.29, *p* = 0.001, *η*_p_^2^ = 0.17, and the proportion of “yes” judgments at 2000 ms (*M* = 0.680, SD = 0.011) was significantly higher than that at 50 ms (*M* = 0.651, SD = 0.012), indicating that exposure time had an impact on the judgment of the mean emotion of faces. The main effect of the group was not significant, *F*(1, 56) = 0.78, *p* = 0.381, *η*_p_^2^ = 0.01.

In summary, this experiment showed that the proportion of “yes” response to mean test faces was significantly higher than those to member and neither test faces, independent of exposure time. Although the mean face was not among the previous set of faces, participants tended to believe that they had seen it before, which is consistent with the previous findings [20, 22]. Remarkably, even when the stimuli were presented for an extremely brief period of 50 ms, both the HDS and LDS groups were still able to make ensemble perceptual judgments of facial crowds. This result suggests an involuntary ability for ensemble perception. This experiment also showed that the HDS group had no difference in implicit ensemble perception of the face set compared to the LDS group when the exposure time was both 2000 and 50 ms. This indicates that the automatic ensemble perceptual ability of the HDS group is not different from that of the LDS group, regardless of exposure time.

#### 4.2.1. General Discussion

Emotional processing and attentional bias associated with depression have long been hot topics. A prevalent view is that people with depression frequently process emotion abnormally and tend to bias attention to negative emotions when viewing a single facial expression (e.g., [52]). The present study aimed to expand on this perspective by examining the ensemble perception of multiple emotional faces simultaneously and investigating the potential influence of depressive tendency on ensemble perception. Study 1 showed that when the faces were exposed for 2000 ms, the PSEs of HDS group were more biased towards anger and their accuracies of explicit ensemble perception were lower than those of LDS group. However, the two groups did not show any difference in either the PSE or accuracy when exposure time was largely shortened. Different from the findings of Study 1, Study 2 demonstrated a different result pattern for implicit ensemble coding. Specifically, HDS group showed no tendency to automatically identify the averaged emotion of multiple faces as a member of a face set compared with LDS group, regardless of exposure time. This suggested that there was no difference between individuals with HDS and individuals with LDS in spontaneous perception of ensemble emotion.

Our Study 1A revealed a negative correlation between the level of depression and the performance of ensemble coding for emotion. For the first time, the present findings illustrated the impact of emotional states on ensemble estimation of facial expressions in individuals with depressive tendencies. Two factors may have contributed to this result. On the one hand, our results can be interpreted by the broaden-and-build theory [53]. According to this theory, positive emotions broaden the scale of attention and increase attentional flexibility, which facilitates global processing. Negative emotions, by contrast, narrow the scale of attention and fixate attention on local visual features, leading to more local processing [54, 55]. We conjecture that the scope of attention of HDS group can be narrowed by depressive tendency—a representative of negative emotions. As a result, local processing takes precedence over global processing, which results in a worse performance in ensemble perception task for HDS group than LDS group. On the other hand, research has provided converging evidence that individuals with depression have an attentional bias to negative stimuli (e.g., [40]). Indeed, the reduced attentional inhibition towards negative stimuli is the main clinical manifestation of impaired cognitive control in depressed patients [56]. They believe that people around them are often unfriendly or hostile [57], so they are reluctant to contact others, stay alone and avoid social interaction [58]. In the long term, this may result in rumination [59]. According to the attentional scope model of rumination, a cognitive pattern similar to the broaden-and-build theory, when depressive states occur, negative stimuli are more likely to be the center of attention and processed repeatedly, while other information outside the attention is ignored [60]. Studies using eye movement techniques have further supported this notion by showing that depressed people spend more time fixating on faces with negative emotion than healthy individuals when presented with various emotional faces [52, 61]. Since ensemble perception can be achieved through sampling a subset of the stimuli [21, 26, 62], depressed individuals are inclined to sample a subset of negative faces to estimate the mean emotion of a crowd. As such, the mean emotion is negatively biased. Notably, while the evidence is derived from studies on clinically depressed patients, the present study extends these interpretations to individuals with depressive tendencies, indicating that the core attentional characteristics associated with negative affect may be shared by the non-clinical depressive trait group.

Our experiments showed that individuals with depressive tendencies exhibit the impaired ability to perceive ensemble emotion only when exposure time was 2000 ms. Additionally, there was greater variance in accuracy when exposure time was 50 ms. This finding is in accordance with prior studies on the processing of single emotional face [24, 38, 39, 63]. Mogg et al. [64], using a dot probe task, found that although depressed patients did not respond faster to negative emotional faces compared with healthy controls, they did spend more time focusing on them [52, 65]. As such, the biases towards negativity are absent during the early stage of information processing or with short exposure times [38, 66], whereas the biases are present when exposure time is lengthened [24, 67]. This makes it difficult for depressed patients to divert their attention away from negative stimuli in later stages of information processing [68, 69]. In our research, when participants had a long time for cognitive processing, depressive tendencies may lead to a prolonged fixation on angry faces. This, in turn, resulted in a negatively biased ensemble perception. Likewise, the variance in accuracy did not stem from differences among the participants, but rather was caused by different exposure times, which reflected the impact of exposure time on the stability of task performances. When exposure time was 50 ms, the stimulus information was difficult to be fully coded, leading to an increase in the randomness of participants' responses, and thus an expansion of variance [24]. In the present study, we observed a similar pattern in individuals with depressive trait tendencies (a non-clinical group), suggesting that the time-dependent emergence of negativity biases may not be unique to clinical depression but also present in subclinical depressive traits.

Recently, [70] found an emotion-amplification effect in crowds—estimating the emotion of a face crowd as more extreme than it actually is. They also found that this effect was more pronounced when exposure time was increased. The study also employed eye tracking, which showed that dwell time on emotional faces was significantly longer than that on neutral faces. This indicated that participants had difficulty disengaging from emotional faces after attending to them. The prolonged exposure time led to attentional bias towards more emotional faces. The present study replicated the emotion-amplification effect in crowds towards negative emotion in both HDS and LDS groups. This result is likely due to the fact that negative expressions have an advantage over positive expressions in attentional capture since they convey potential threats [71, 72]. Thus, participants may find it harder to disengage from angry expressions than from happy expressions. Furthermore, the present study demonstrated that emotion-amplification effect in crowds was significantly greater in HDS group than in LDS group when presentation time was 2000 ms. This could be attributed to the fact that compared to LDS group, individuals with HDS might have a higher tendency to focus on angry faces due to their depressive state. Angry faces were then given more weight in the formation of working memory, which could bias the estimate of averaged representation [73]. As a result, the amplification effect of crowd emotion was intensified in individuals with HDS when the exposure time was longer.

Ensemble perception can be achieved explicitly or implicitly, which may involve distinct neural pathways [74]. To investigate if the depressive tendency would bias both forms of ensemble coding, we examined the explicit ensemble perception using the mean discrimination paradigm (Study 1) and the implicit ensemble perception using the membership identification task (Study 2). Consistent with Hansmann-Roth et al. [74], the present study also showed a different result that depressive tendency could bias emotional processing in the explicit task but not in the implicit task. Notably, in Study 2, participants were not explicitly asked to perceive emotion ensembles, yet the results still suggested that they did so, which supported the automatic representation view of average emotion [10, 20, 22]. These findings suggest that depressive tendency can bias ensemble perception of emotion at explicit level but not at implicit level, which indicates that explicit and implicit ensemble perceptions are two different informational processing pathways.

The present study has several limitations that need to be addressed. First, all of the faces utilized in the experiments were derived from a single male model, which may induce bias and learning effect. In addition, given established gender effects on emotion perception (e.g., [75]), using both male and female models would be preferable. Future studies should investigate ensemble perception of facial expressions using different identities with both male and female to minimize potential biases and increase ecological validity. Second, it is important to note that depressive tendencies, as a milder form of depression, are not equivalent to depression [76]. Hence, our findings are restricted to non-clinical individuals with high levels of depressive tendency. Future research may examine whether our findings can be generalized to clinical population diagnosed with depression and explore potential interventions to reverse the adverse cognitive bias in these individuals. Third, participants in Study 1 were asked to judge whether the test face was angrier than the preceding face set. Our results showed a significant advantage in recognizing angry expressions compared to happy expressions, indicating a genuine processing difference between the two. Though this task follows the typical methodology used by previous research (e.g., [49]), it is important to note that the asymmetric question of asking only “angrier” may introduce a systematic response bias (i.e., judging ambiguous faces as angrier). Therefore, even though Study 1 showed that the PSE of both HDS and LDS groups was significantly smaller than 0 at exposure times of 50 and 2000 ms, we must be cautious in concluding that this reflects a genuine perceptual bias toward anger. To eliminate this potential bias and confirm whether such a perceptual bias exists, future research could adopt a balanced design: in half of the trials, participants could judge whether the test face appears angrier than the face set, and in the other half, whether it appears happier.

### 4.3. Conclusion

The current research demonstrates that depressive tendency can bias ensemble coding for emotional faces at explicit level but not at implicit level. High levels of depressive tendency are associated with reduced performance in explicit ensemble perception and a tendency to negatively estimate ensemble emotions. However, this bias becomes apparent only when exposure time for faces is 2000 ms. These findings provide insight into the perceptual characteristics of individuals with depressive tendencies when viewing face crowds and contribute to current theories regarding emotional processing in depression from the perspective of ensemble perception.

## Data Availability

The data and materials for the experiments reported here are available on the Open Science Framework at https://osf.io/8aycd/?view_only=458bb68ecbf046ab9f69a4080fa72336.
